# Higher Circulating Neutrophil Counts Is Associated with Increased Risk of All-Cause Mortality and Cardiovascular Disease in Patients with Diabetic Kidney Disease

**DOI:** 10.3390/biomedicines12081907

**Published:** 2024-08-20

**Authors:** Ruiyan Xie, David M. Bishai, David T. W. Lui, Paul C. H. Lee, Desmond Y. H. Yap

**Affiliations:** 1 Division of Nephrology, Department of Medicine, Queen Mary Hospital, The University of Hong Kong, Hong Kong SAR 999077, China; 2 Division of Health Economics, Policy and Management, School of Public Health, LKS Faculty of Medicine, The University of Hong Kong, Hong Kong SAR 999077, China; 3 Division of Endocrinology and Metabolism, Department of Medicine, Queen Mary Hospital, The University of Hong Kong, Hong Kong SAR 999077, China; dtwlui@hku.hk (D.T.W.L.);

**Keywords:** neutrophils, diabetic kidney disease, cardiovascular, mortality

## Abstract

Background: Accumulating evidence has suggested the pathogenic roles of chronic inflammation and neutrophils in diabetic kidney disease (DKD). This study investigated the relationship between neutrophils, all-cause, and cardiovascular disease (CVD) mortality in type 2 diabetes mellitus (T2DM) patients with DKD. Methods: We used data from the National Health and Nutrition Examination Surveys (NHANES) from 2005 to 2020 to investigate the relationship between circulating neutrophils counts, kidney function indices, all-cause, and CVD mortality in adult T2DM patients with DKD. Clinical predictive models and risk scores for long-term mortality were constructed. Results: 44,332 patients [8034 with T2DM and 36,323 without T2DM] were included. Two thousand two hundred twenty patients had DKD, and 775 died (31.5% related to CVD) during a follow-up of 6.18 (range: 5.94–6.42) years. Higher neutrophil counts (Quartile 4, Q4) were associated with increased all-cause and CVD mortality [HR 1.73 (95% CI 1.34–2.25) and 1.81 (95% CI 1.14–2.89), respectively, *p* < 0.0001 and 0.01]. Neutrophil counts in Q4 showed a positive correlation with urine albumin-creatinine ratio (UACR) but a negative association with eGFR (*p* < 0.01 for all). Clinical predictive models incorporating neutrophil counts showed satisfactory performance in forecasting 5-year and 10-year CVD mortality-free survival (ROC AUC 0.824 and 0.842, respectively), and the nomogram-predicted survival demonstrated good concordance with observed survival. Conclusions: Higher levels of circulating neutrophil counts show a significant correlation with renal abnormalities and higher all-cause and CVD mortality in T2DM patients with DKD. The novel clinical predictive models and risk scores incorporating neutrophil counts may facilitate stratification and, hence, risk factor management in DKD patients.

## 1. Introduction

Diabetes mellitus (DM) is a major global healthcare burden. As of 2021, the estimated prevalence of DM patients between the age of 20–79 is 537 million, and the numbers will continue to rise and reach 780 million in 2045 [1]. DM is associated with various macro- and microvascular complications. Diabetic kidney disease (DKD) is an important microvascular complication in DM patients and represents the most common cause of chronic kidney disease (CKD) and end-stage kidney disease (ESKD) in the United States and many other localities [2,3,4]. Cardiovascular disease (CVD) is the leading cause of mortality in CKD and ESKD patients and is also a serious macrovascular complication of DM [5,6]. 

The interaction between DM, CKD, and CVD is highly complex. DM is a major risk factor for CVD and induces a myriad of cellular, epigenetic, and post-translational alterations that affect the health of vasculatures [7]. These changes include glucose-related tissue toxicity, endothelial dysfunction, histone hypermethylation, DNA methylation, chronic inflammation and thrombosis, mitochondrial metabolism, atherogenic dyslipidemia, and arterial hypertension, all of which escalate the risk of CVD [8,9,10]. CKD aggravates atherosclerosis and CVD via the detrimental effects of uremic toxins and hypertension on vascular endothelial cells, accelerated vascular calcification, increased pro-thrombotic states, and chronic inflammation [11]. Indeed, the presence of CKD compounds the risk of CVD in DM patients, as the presence of DKD in type 2 DM (T2DM) patients is associated with a significantly escalated risk of CVD morbidity and mortality compared to T2DM patients without renal involvement [11]. In this context, the synergistic effects of DM and CKD on CVD pathogenesis remain elusive. An improved understanding of these pathogenic mechanisms may potentially help retard renal progression and prevent adverse cardiovascular outcomes in DM patients.

While DM is often recognized as a metabolic disorder, growing evidence has suggested that chronic inflammation assumes crucial pathogenic contributions in its macro- and microvascular complications [12,13,14]. Neutrophils constitute the largest portion of white blood cells and represent a major component of immunity. The immune response mediated by neutrophils is strongly associated with oxidative stress, neutrophil extracellular traps (NETs), extracellular vesicles, and inflammasome activation [15,16], which all have been implicated in the pathogenesis of CVD [17,18]. Furthermore, neutrophil count is a significant indicator of DKD in autoimmune diabetes [19]. Previous studies showed that activated circulating neutrophils were associated with the development of type 1 diabetes mellitus (T1DM) [20]. One recent study also indicated that neutrophil count was positively associated with CKD development in Chinese patients with diabetes [21]. Previous literature has reported the correlations between neutrophils, cardiovascular inflammation, and outcomes of cardiometabolic diseases [22,23]. For instance, the elevated neutrophil count was linked to an overall increased incidence of coronary events and CV death, especially in patients with ST-elevation myocardial infarction [24]. However, the relationship of circulating neutrophil counts with CVD and all-cause mortality in patients with T2DM and DKD remains unknown. 

Recognizing the importance of neutrophilic response in CVD and DM, we hypothesize that higher neutrophil counts in T2DM patients with DKD are associated with increased risk of all-cause and CVD mortality. Here, we used data from a large-scale prospective cohort from the NHANES database to test our hypothesis. Clinical predictive models that incorporated circulating neutrophil counts were also constructed for prognostication of long-term patient outcomes in T2DM patients with CKD.

## 2. Materials and Methods

### 2.1. Study Population and Definitions of the Exposure and Outcome Variables

The National Center for Health Statistics (NCHS) in the United States initiated the National Health and Nutrition Examination Survey (NHANES), which is a large, population-based, repeated cross-sectional survey (https://www.cdc.gov/nchs/index.htm; accessed on 1 May 2023). The NHANES gathers information on the health and nutrition through personal interviews of participants at their homes, mobile examination centers, and laboratories across the country.

In this study, the exposure variable was circulating neutrophil count in adult type 2 DKD patients. The study outcome was kidney functions, CVD mortality, and all-cause mortality. The confounding factors were those could affect neutrophils count and mortality. Therefore, we extracted the data of circulating neutrophils count, kidney functions such as urine albumin, urine creatinine, lifestyles like drinking and smoking, and confounding factors such as hypertension and hyperlipidemia. The exclusion criteria included missing data, type one DM patients, age less than 18.

We first used data from seven NHANES cycles data: 2005–2006, 2007–2008, 2009–2010, 2010–2012, 2013–2014, 2015–2016, and 2017–2020, which included indicators of T2DM and kidney functions [estimated glomerular filtration rate (eGFR), urine albumin and creatinine]. At first, we collected a total of 85,750 participants’ information (age, gender, and race), then selected those age older than 18 years (*n* = 49,461) who reported to the mobile centers. Of the 49,461 participants, those with missing data on hematologic laboratory values (*n* = 2654), eGFR (*n* = 890), UACR (*n* = 695), T2DM diagnosis (*n*= 728), and aspirin use (*n* = 36) were excluded. We also excluded patients with T1DM diagnosis because these patients have strong autoimmune components, which would confound our results [19]. After the exclusion of patients with T1DM (*n* = 126), the data of 44,332 patients (8034 with T2DM and 36,298 without T2DM) were used for analysis. 

**Figure 1:**
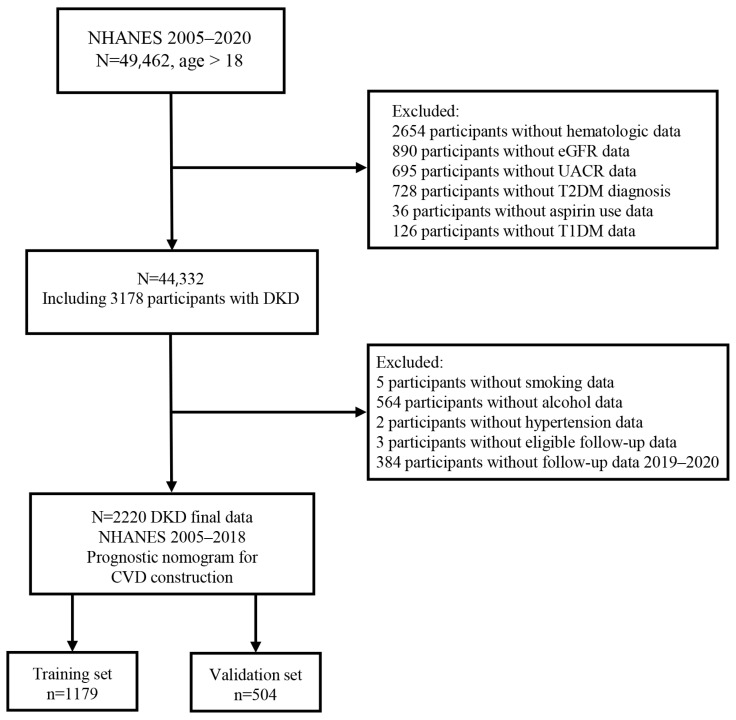
The flow chart of this study.

To investigate the relationship of neutrophil count with all-cause and CVD mortality further, we selected the data from NHANES 2005–2018. The cause of death was defined using the National Death Index records, and deaths with ICD-10 codes (I00–I09, I11, I13, I20–I51) were regarded as CVD deaths. The CVD death coding specifically referred to deaths related to rheumatic heart diseases, hypertensive heart, ischemic heart disease, and heart failure. Out of the 3178 DKD participants, we excluded those with missing values on smoking status (*n* = 5), alcohol use (*n* = 564), hypertension (*n* = 2) and follow-up data (*n* = 3). Data from 2019–2020 (*n* = 384) were also excluded because there was no follow-up information on CVD outcomes. Therefore, a total of 2220 DKD participants were included in the final analysis (Figure 1).

### 2.2. Definition of DM

The diagnostic criteria for T2DM in this study were defined as patients being told by attending physicians that they have DM; or HbA1c (%) ≥ 6.5; or fasting glucose ≥ 7.0 mmol/L; or random blood glucose ≥ 11.1 mmol/L; or two-hour oral glucose tolerance test (OGTT) blood glucose ≥ 11.1 mmol/L; or use of anti-diabetic medications including insulin [1]. Patients with gestational DM were excluded. In this study, we excluded all patients with T1DM. 

### 2.3. Definition of CKD

The eGFR was calculated using the CKD-EPI creatinine equation (2009). The urine albumin-creatinine ratio (UACR) was the ratio of urine albumin concentration to urine creatinine concentration. CKD was defined as UACR ≥ 30 mg/g or/and eGFR < 60 mL/min per 1.73 m^2^, according to the KDIGO 2021 clinical practice guideline [16].

### 2.4. Definition of Smoking and Alcohol Use

“Smoking” was defined as ‘yes’ (smoked > 100 cigarettes in life and smoked some days or every day) or ‘no’ (never smoked, or <100 cigarettes in life or smoked > 100 cigarettes in life and smoke not at all now). “Alcohol use” was defined as ‘yes’ (had ≥ 12 drinks in one year) or ‘no’ (had ≥ 12 drinks in 1 year and did not drink last year or did not drink last year but drank ≥ 12 drinks in a lifetime or had < 12 drinks in a lifetime).

### 2.5. Definition of Hypertension and Hyperlipidemia

Hypertension was defined as three or more consecutive systolic blood pressures ≥ 140 mm of mercury (mmHg) or above, diastolic blood pressures ≥ 90 mmHg, or the use of anti-hypertensive medication. Hyperlipidemia was defined as serum triglyceride (TG) ≥ 150 mg/dL; total cholesterol (TC) ≥ 200 mg/dL or low-density lipoprotein (LDL) cholesterol ≥ 130 mg/dL or high-density lipoprotein (HDL) cholesterol < 40 mg/dL(male)/50 mg/dL(female); or use of lipid-lowering medication.

### 2.6. Other Clinical Parameters and Covariates

The degree of neutrophilic inflammation was gauged by the neutrophil-to-lymphocyte ratio (NLR) and systemic immune-inflammation index (SII) [(neutrophil counts × platelet counts) ÷ lymphocyte counts]. As previous studies suggested, age, sex, race, smoking status, alcohol use, hypertension, and hyperlipidemia were associated with kidney outcomes and neutrophils count [25,26]. Hence, these parameters were collected at baseline using standardized interview questionnaires and adjusted as co-variates in the regression models.

### 2.7. Statistical Analysis

The baseline patient characteristics were presented as frequency (percentages) and mean ± standard errors (SEs). The data from 2005–2020 or 200–2018 were adjusted using the sample weights, strata, and primary sampling units to generate more accurate national estimates. Categorical variables were compared using Chi-square tests. Continuous variables with normal distribution were analyzed with one-way ANOVA, while variables with non-normal distribution were analyzed by the Kruskal–Wallis test. The data were also analyzed according to different quartiles of neutrophil counts (Q1–Q4). 

Weighted Cox proportional hazard regression models were used to examine the relationships between different quartiles of neutrophil counts, all-cause mortality, and survival from CVD mortality. The crude model was not adjusted for any covariates. Model 1 was adjusted for age, race, and sex. Model 2 was adjusted for smoking status, alcohol use, hypertension, hyperlipidemia, eGFR, and UACR in addition to the factors in Model 1. Model 3 was further adjusted for aspirin usage. The linear trend was analyzed based on the mean value in each category. We also investigated the associations of neutrophil count levels with CVD and all-cause mortality in a restricted cubic spline (RCS) analysis of count-response with weighted adjustment in Model 3 (using the “rms” *R* package 4.3.2). A *p* value of <0.05 (two-sided) was considered as statistically significant.

The variables in Model 2 were further used to construct the clinical model predicting 5-years and 10-years overall survival (OS) and survival free of CVD mortality in patients with DKD using “foreign”, “survival”, “caret” and “rms” R packages. First, we did the survival data-splitting of 2220 DKD participants and set the random seed number using 200. The data were divided into development and validation cohorts in the proportion of 7:3. The clinical information of 1179 participants with DKD was used for the development cohort of CVD mortality-free survival, while data from the remaining 504 DKD patients were used for the validation cohort. The two cohorts were all performed under 100 bootstrap resamples. We also constructed a clinical predictive model and nomogram for overall survival in DKD patients. Cox proportional hazard regression methods were used to determine the risk scores for CVD mortality-free survival. The risk scores were divided into low- or high-risk groups according to the median value of risk scores. Receiver operating characteristic (ROC) curves were constructed to evaluate the model performance using “timeROC”, “survminer” and “survival” *R* package 4.3.2. All statistical analyses were performed by the “Survey” package using *R* package 4.3.2 and GraphPad Prism 9 software.

## 3. Results

### 3.1. Characteristics of the Study Population

The data for 44,332 participants aged ≥ 18 years were extracted from NHANES database, of which 18% individuals had T2DM. The baseline prevalence of CKD was 40% in T2DM patients, while the prevalence of CKD was 13.0% in those without DM. The DKD group showed the highest baseline white cell counts and neutrophil counts, percentage, neutrophil to lymphocyte ratio (NLR), and systemic immune-inflammation index (SII) compared to DM patients without CKD, non-DM patients with CKD and non-DM patients without CKD (Table 1). The DKD group also showed the worst eGFR level and UACR among the four groups (Table 1). The DKD had higher rates of anti-hypertensive, angiotensin-converting enzyme inhibitors (ACEI), and angiotensin ii inhibitors (ARB), anti-diabetic drugs, lipid-lowering agents, and aspirin compared to other groups (Table 1). Amongst DKD patients, rates of ACEI and ARB use were 39.52% and 21.02%, respectively.

### 3.2. Association between Neutrophils Count and Kidney Function in Patients with DKD

Circulating neutrophils percentage, but not absolute count, was inversely correlated with eGFR (Table 2). NLR and SII also showed inverse relationships with eGFR. Circulating neutrophil counts and percentage and SII all showed a positive cross-sectional correlation with UACR in DKD patients (*p* < 0.05) (Table 2). When stratified into different quartiles of neutrophil counts (Supplementary Table S1), Q2, Q3, and Q4 all showed significant positive correlations with UACR but negative associations with eGFR (Table 3). The results were consistent after adjustment of patient characteristics, including age, sex, race, smoking, and alcohol status, as well as hypertension and hyperlipidemia.

### 3.3. Associations between Neutrophil Counts, All-Cause Mortality and CVD Mortality

A total of 2220 DKD patients from NHANES 2005–2018 were followed for a median of 6.18 (range: 5.94–6.42) years. Two hundred thirty-eight patients (10.72%) had underlying CVD, and 87 (3.9%) patients received aspirin at baseline. There were 775 deaths from all causes, of which 238 (30.71%) were related to CVD, and 131 (16.90%) were due to malignancy. Neutrophil counts in Q4 showed a significant association with all-cause mortality and CVD mortality [HR 1.73 (95% CI 1.34–2.25) and 1.81 (95% CI 1.14–2.89), respectively, *p* < 0.0001 and 0.01] (Table 4)]. The results were consistent using different regression models with adjustments for patient demographics, smoking status and alcohol use, hypertension, hyperlipidemia, eGFR, UACR, and use of aspirin.

**Figure 2:**
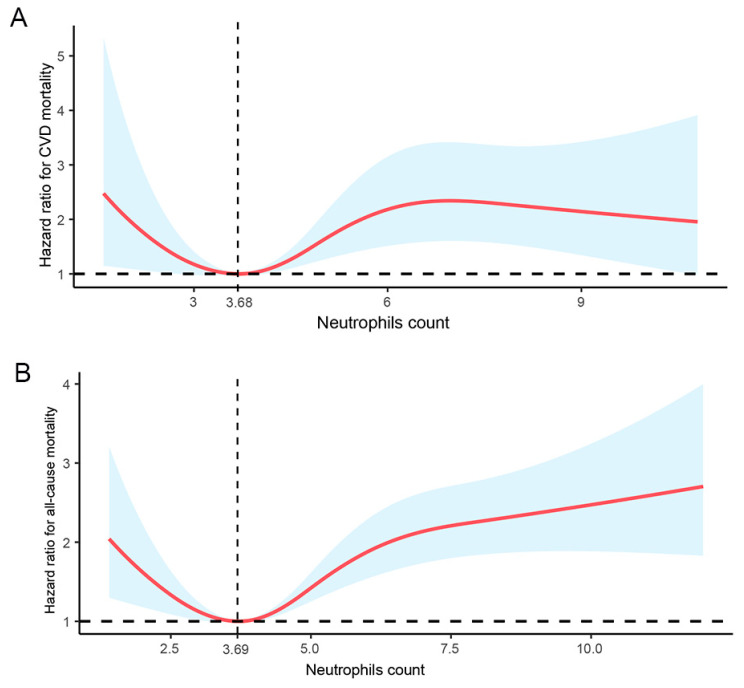
Dose-response relationship between circulating neutrophil counts of DKD patients and (**A**) CVD mortality and (**B**) all-cause mortality. Adjusted for age, sex, race, smoking, alcohol use, hypertension, hyperlipidemia, eGFR, and UACR in a logistic regression with the RCS model. The shaded area represents the estimated relative risk and the 95% CI. The vertical line represents cut-off value, and the horizontal dashed line represents reference line of no association is indicated at a hazard ration of 1.0. CI, confidence interval. *p* non-linearity = 0.0029 (CVD mortality) and 0.0000 (all-cause mortality), respectively.

In the multivariate logistic regression analysis with RCS, circulating neutrophil counts showed a nonlinear association with CVD mortality (Figure 2A, *p* = 0.0029) and all-cause mortality (Figure 2B, *p* = 0.0000) after adjusting for multiple confounding factors. Neutrophil counts of 3.68 × 10^9^/L and 3.69 × 10^9^/L were associated with the lowest risk of CVD mortality and all-cause mortality, respectively, in DKD patients.

### 3.4. Development and Validation of a Predictive CVD and All-Cause Mortality Risk Nomogram

**Figure 3:**
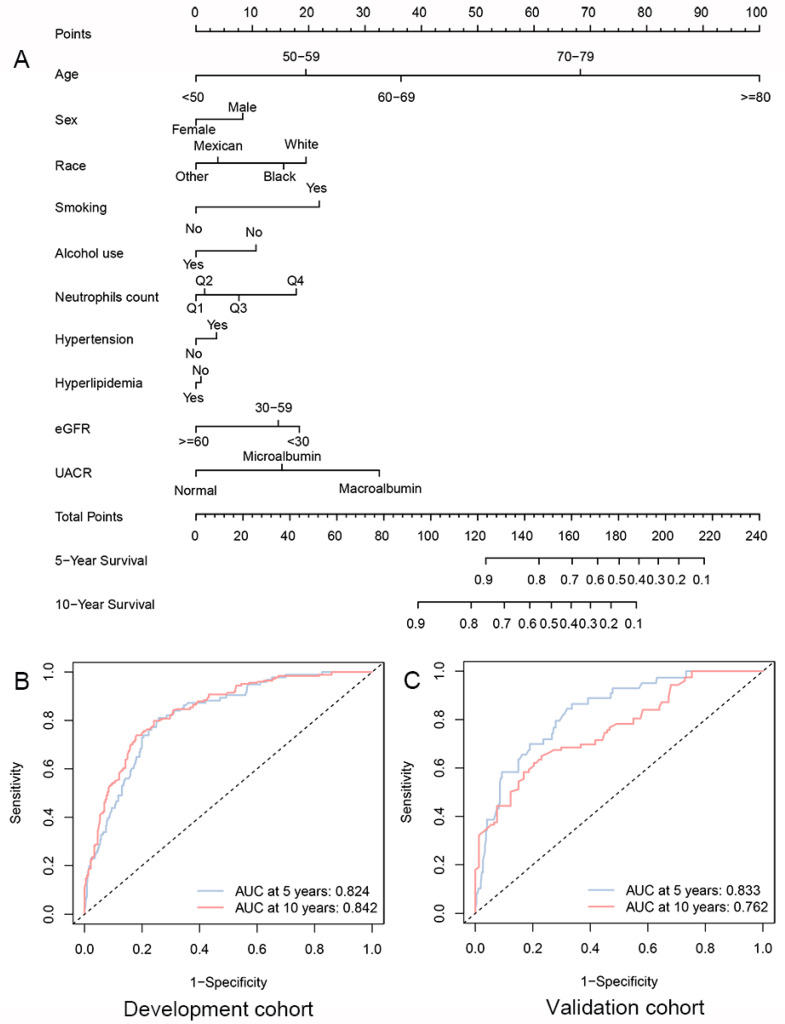
Clinical predictive models for long-term CVD mortality-free survival among individuals with DKD. (**A**) Nomogram for predicting 5- and 10-year CVD mortality-free survival between DKD patients in the development cohort. ROC curves of the predictive nomogram in (**B**) development and (**C**) validation cohorts. Q1, Quartile 1; Q2, Quartile 2; Q3, Quartile 3; Q4, Quartile 4.

Based on the strong correlation of circulating neutrophils count with CVD mortality of DKD individuals in Model 2, ten factors of Model 2 were selected to establish a clinical predictive nomogram. The nomogram incorporated ten lines to determine the corresponding points. The sums of these points could be calculated and predict the 5- and 10-years survival free of CVD in DKD patients (Figure 3A and Tables S2 and S3).

The time-dependent ROC analysis showed that the model had good performance for predictive 5-year and 10-year CVD mortality-free survival [Development cohort: AUC 0.824 and 0.842, respectively, for 5-year and 10-year CVD mortality-free survival (Figure 3B); Validation cohort: AUC 0.833 and 0.762, respectively, for 5-year and 10-year mortality-free survival (Figure 3C)]. Cox regression analysis showed that the concordance indices (C-index) of the development and validation cohorts were 0.805 and 0.801, respectively. The calibration curve showed that the model-predicted results were in good agreement with the actual results, especially for the 5-year results (Figure S4). Similar to the results for overall survival, the clinical predictive model showed good performance for portending overall survival in DKD patients (Tables S5 and S6). 

**Figure 4:**
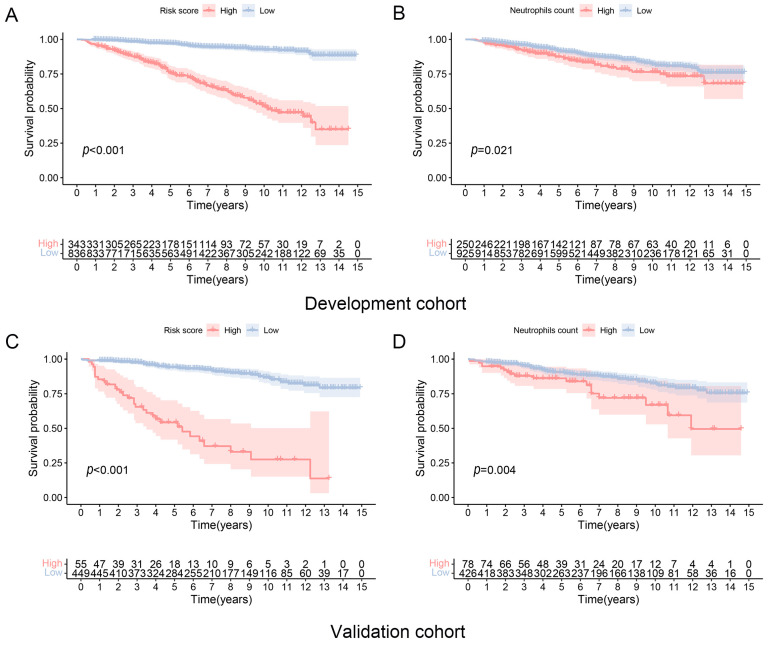
CVD-free mortality in patients with diabetic kidney disease according to risk scores and baseline neutrophils counts. (**A**,**B**) Development cohort and (**C**,**D**) Validation cohort.

**Figure 5:**
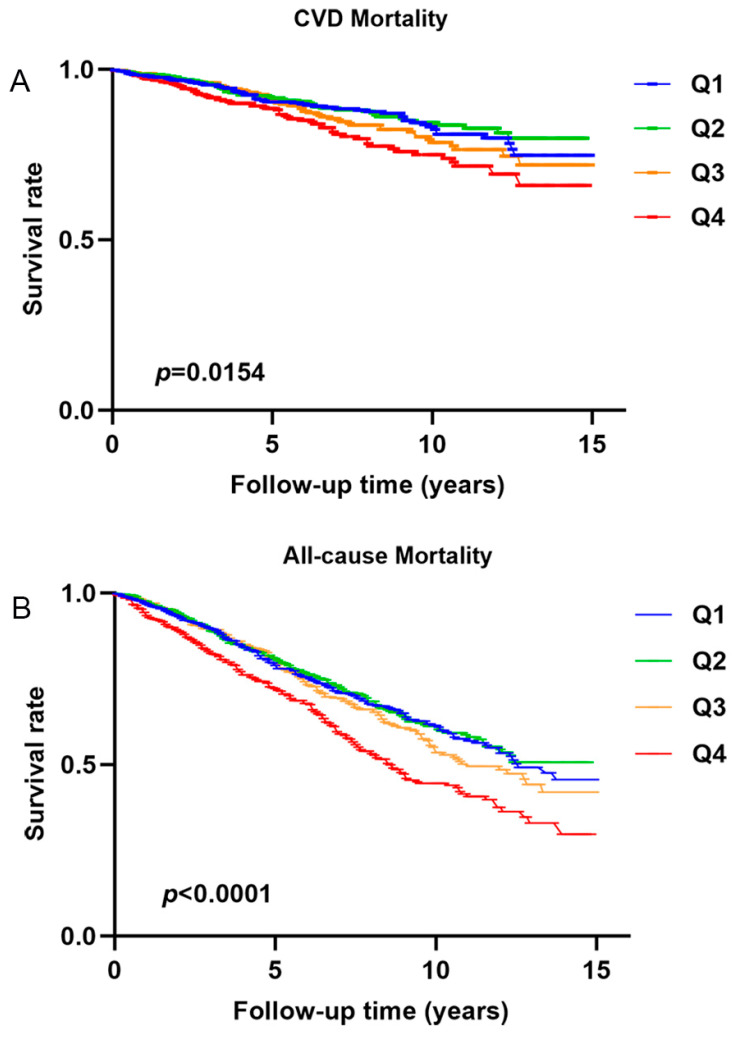
Survival is free of (**A**) cardiovascular disease mortality and (**B**) all-cause mortality in diabetic kidney disease patients with different quartiles of neutrophil counts.

DKD patients with higher risk scores showed worse CVD mortality-free survival compared to the lower risk scores group (Figure 4A,B). DKD patients with higher neutrophil counts (≥5.70 × 10^9^/L) also showed inferior CVD mortality-free survival than DKD patients with lower neutrophil counts (<5.70 × 10^9^/L) (Figure 4C,D). Similar to the results for CVD mortality-free survival, DKD patients with higher risk scores and neutrophil counts (≥5.40 × 10^9^/L) also showed worse overall survival than those with lower risk scores and lower neutrophil counts (<5.40 × 10^9^/L) (Figures S7 and S8). The CVD mortality-free survival model, which incorporated the use of aspirin on top of Model 2, showed similar results to the model without aspirin use (Figures S9 and S10). Furthermore, DKD patients with neutrophil counts in Q4 showed lower overall survival and CVD mortality-free survival compared to patients with neutrophil counts in other quartiles (*p* < 0.001 and 0.0154, respectively) (Figure 5A,B).

## 4. Discussion

Our results suggest that circulating neutrophil count is associated with all-cause and CVD mortality in DKD. Furthermore, clinical predictive models that incorporated neutrophil counts for forecasting long-term survival showed high AUC in the ROC curve. In addition, circulating neutrophil counts also showed a correlation with proteinuria and renal function in DKD patients. These findings are clinically important as they may help identify high-risk subjects with DKD who may require more aggressive cardiovascular risk factor control and reno-protection.

In this study, circulating neutrophil counts showed a nonlinear relationship with the risk of death. Higher circulating neutrophil counts, in particular, are an associated risk factor for all-cause and CVD mortality. One clinical study suggested that the CKD risk was increased in T2DM patients when the circulating neutrophil count exceeded 3.6 × 10^9^/L [21]. The minimum value of neutrophil count was similar to that in this study. On the one hand, while DKD and CVD are conventionally viewed as metabolic and vascular disorders, growing evidence has implicated the pivotal role of chronic inflammation in renal progression and acceleration of atherosclerosis. On the other hand, the presence of DM and CKD can also induce chronic inflammation that aggravates other end-organ damage [27]. Our observations suggest that higher neutrophil counts, reflecting an increased chronic inflammatory state, contributed to escalated all-cause and CVD mortality. Indeed, neutrophils are the most abundant leucocytes in humans, and neutrophil-induced inflammation has long been recognized as a critical factor in the pathogenesis of CVD. Variations in circadian rhythms and disease-specific microenvironments contribute to the heterogeneity of neutrophils. These neutrophils are activated by a range of stimuli, including chemokines, cytokines, and dam-age-associated molecular patterns. Once activated, neutrophils facilitate the inflammatory response in CVD, including neointimal formation, myocardial infarct, heart failure, and stroke, through mechanisms such as degranulation, phagocytosis, the generation of reactive oxygen species (ROS), and the release of NETs. Notably, NETs play a significant role in promoting immuno-thrombosis through their interactions with various cellular and molecular components. Other hallmark functions of neutrophils, such as phagocytosis, degranulation, and generation of ROS, are pertinent to the immunopathogenesis and repair in various forms of CVD [22,28]. Atherosclerosis, an initiating event in cerebrovascular and CVD is strongly driven by inflammatory processes on top of lipid infiltration and altered vascular stress. Evaluating the severity and vulnerability of atherosclerotic plaques is essential for the effective prevention and management of CVD [29]. DKD could accelerate the development of atherosclerotic plaques. One study suggested that neutrophils could exacerbate tissue damage and induce inflammation in advanced atherosclerosis by triggering smooth muscle cell (SMC) lysis and death [30]. Neutrophils are regarded as regulators of cardiovascular inflammation and mortality [31]. Our study indicated that neutrophils may be an independent risk factor for CVD or all-cause mortality in DKD patients after adjusting confounding factors such as hypertension and hyperlipidemia. To take one step further, our result may fuel future studies to examine patients with atherosclerotic plaque associations to better understand the broader implications of our findings. The association between lower neutrophil counts and mortality may be explained by malnutrition.

This study also implicated a relationship between neutrophil counts and the pathology of DKD, as accumulating data have described the role of chronic inflammation in the development and progression of DKD [32,33]. In line with another cross-sectional study, we observed circulating neutrophils correlated positively with proteinuria but negatively with eGFR in DKD patients [21]. Here, we observed a very strong positive relationship between neutrophils and UACR. As micro- and macro-albuminuria are early events in the disease course of DKD, our observations insinuated the role of chronic inflammation related to neutrophilic activity in the development and progression of DKD. The association between systemic inflammatory indices (NLR and SII), UACR, and eGFR in this cohort further supported our postulation [34,35]. In addition, molecular expression in neutrophils may affect the progression of DKD, though limited studies have been reported. One research indicated that neutrophil counts were closely associated with DKD in patients with auto-immune diabetes, indicating that neutrophil-mediated inflammation may be involved in the pathogenesis of DKD [19]. Single-cell RNA transcriptomic analysis has revealed that neutrophil count is higher in the kidney tissue of the DKD rat model compared to the normal group [36]. Molecular and genetic studies indicate that podocytes and endothelial cells are the crucial points to drive albuminuria and early DKD. Cellular crosstalk has been indicated among neutrophils with podocytes and endothelial cells [37]. Putative pathogenic mechanisms of how neutrophils may aggravate DKD include the release of NETs, upregulation of pyroptosis-associated proteins, activation of NLRP3 inflammasome in glomerular endothelial cells, and secretion of neutrophil gelatinase [38,39,40]. Our findings are clinically relevant because some emerging treatments for DKD may also attenuate neutrophil-related inflammatory pathways. For instance, pentoxifylline (an anti-inflammatory agent) may confer additional benefits for DKD patients via reduction of neutrophil degranulation and endothelial leukocyte adhesion [41]. 

Based on the correlation between circulating neutrophils and mortality, we constructed clinical predictive models and risk scores that incorporate neutrophil counts to prognosticate long-term patient survival. One merit of our predictive models is the use of readily available clinical parameters, including patient demographics and neutrophil counts. The predictive models showed respectable performance in portending long-term survival in DKD patients (especially for 10-year mortality) when compared with other predictive models [42,43]. The nomogram-predicted survival using this model in DKD patients also demonstrated good concordance with observed patient survival. Our data also indicated that patients in the high-risk score group had worse long-term survival compared with those in the low-risk score group. One should appreciate that neutrophil counts can be significantly affected by infection, and therefore, the neutrophil counts used in a predictive model/risk score calculation should be measured at a time when the patient is free from infection. These predictive models and risk scores are clinically useful because they provide a convenient means to forecast long-term mortality in DKD patients and guide appropriate strategies for CV risk factor control in these individuals.

## 5. Strengths and Limitations

One important limitation of this study was its cross-sectional design, where all clinical parameters used for analysis were single-timepoint values measured at baseline. Also, some long-term follow-up data on renal parameters and outcomes are lacking, and hence, we cannot adequately assess the relationship between neutrophils, CKD progression, and the development of ESKD. One should appreciate that our study spanned a relatively long period of time (2005–2018), during which there was substantial progress in medical treatments and sensitivities of clinical tests. For instance, while sodium-glucose cotransporter two inhibitors (SGLT2i) have demonstrated robust protective effects on adverse cardiorenal outcomes, such data were only available in NHANES since 2013, and hence the relationship between SGLT2i and cardiorenal outcomes cannot be adequately examined [only 15 DKD patients had data on SGLT2i]. In addition, some other important confounding factors that affect neutrophil counts (e.g., infection status) were not available from the NHANES database. Additionally, the robustness of the predictive model was robust can be better validated using other approaches, such as the k-fold cross-validation test. In this study, the predictive models were largely derived from clinical parameters, and it remains to be tested whether the incorporation of molecular/omics information may enhance the performance of our models. Lastly, demographic data obtained from questionnaires might potentially be inaccurate or incomplete, and the diagnoses of DKD in T2DM patients were not biopsy-confirmed. Notwithstanding, our results were derived from a well-established multi-ethnic national survey, which had been extensively and reliably used to generate scientific research and outputs. Despite spanning a long study duration (NHANES 2005–2020), key kidney function indices (e.g., UACR and eGFR) at baseline, as well as information on all-cause and CVD mortality, were comprehensively available for analysis. These data enable us to properly assess the relationship between neutrophils and mortality in T2DM patients with DKD and construct useful clinical tools to predict long-term mortality risk. 

## 6. Conclusions

Higher levels of circulating neutrophil counts show a significant correlation with renal abnormalities and higher all-cause and CVD mortality in T2DM patients with DKD. The novel clinical predictive models and risk scores incorporating neutrophils count may help risk stratification in DKD patients and hence institute appropriate risk factor management.

## Figures and Tables

**Table 1:** Baseline characteristics of participants from NHANES 2005–2020.

		No Diabetes Mellitus		Diabetes Mellitus		
Variable	Total	No CKD (*n* = 31,591, 77%)	CKD (*n* = 4707, 9.3%)	No CKD, (*n* = 4856, 8.7%)	CKD, (*n* = 3178, 5.0%)	*p* Value
Predictive population	224,500,575	173,094,552	20,850,397	19,558,373	10,997,253	
Age	46.79(46.37,47.22)	43.21(42.81,43.60)	57.91(57.12,58.70)	56.73(56.18,57.29)	64.51(63.76,65.27)	<0.0001
Sex, %						<0.0001
Female	51.04	50.25	61.55	48.35	48.38	
Male	48.96	49.75	38.45	51.65	51.62	
Race, %						<0.0001
White	66.55	66.92	71.89	60.59	61.25	
Black	10.73	10.19	10.87	13.48	14.11	
Mexican America	8.78	8.86	6.32	9.99	9.91	
Other	13.93	14.02	10.92	15.93	14.73	
BMI	29.01(28.86,29.15)	28.32(28.18,28.46)	28.74(28.48,29.00)	33.18(32.85,33.52)	32.95(32.52,33.38)	<0.0001
WBC (×10^9^/L)	7.27(7.22,7.32)	7.14(7.10,7.19)	7.46(7.30,7.63)	7.68(7.59,7.77)	8.04(7.91,8.17)	<0.0001
Neutrophils (×10^9^/L)	4.29(4.25,4.32)	4.19(4.16,4.23)	4.42(4.35,4.49)	4.60(4.53,4.67)	4.98(4.89,5.07)	<0.0001
Neutrophils percentage (%)	58.16(57.97,58.35)	57.68(57.49,57.88)	59.64(59.26,60.03)	59.04(58.61,59.47)	61.30(60.82,61.78)	<0.0001
Lymphocytes (×10^9^/L)	2.16(2.14,2.18)	2.15(2.14,2.17)	2.20(2.07,2.33)	2.23(2.19,2.28)	2.14(2.07,2.22)	<0.001
Hb	14.26(14.22,14.30)	14.34(14.30,14.39)	13.84(13.77,13.91)	14.20(14.13,14.27)	13.82(13.73,13.91)	<0.0001
PLT (×10^9^/L)	248.28(246.86,249.70)	249.70(248.24,251.16)	243.38(240.42,246.34)	246.01(242.88,249.14)	239.22(235.17,243.28)	<0.0001
NLR	2.17(2.15,2.20)	2.10(2.07,2.12)	2.44(2.39,2.49)	2.29(2.23,2.34)	2.68(2.61,2.76)	<0.0001
SII	540.73(534.54,546.91)	526.03(519.46,532.60)	593.21(577.37,609.05)	559.33(544.72,573.94)	639.47(619.10,659.84)	<0.0001
Albumin, urine (mg/L)	33.76(31.51,36.01)	9.13(8.94, 9.31)	121.29(106.82,135.75)	11.93(11.55, 12.31)	294.34(258.23,330.45)	<0.0001
Creatinine, urine (mg/dL)	122.91(121.43,124.38)	125.05(123.36,126.75)	112.94(110.16,115.71)	120.33(117.19,123.46)	112.61(109.21,116.00)	<0.0001
UACR (mg/g)	33.36(30.96,35.76)	7.51(7.41, 7.61)	125.39(108.45,142.32)	10.17(9.95, 10.39)	306.94(269.23,344.65)	<0.0001
eGFR (mL/min/1.73 m^2^)	95.04(94.47,95.61)	99.27(98.76,99.79)	76.14(74.86,77.42)	91.75(91.04,92.45)	70.16(68.69,71.63)	<0.0001
Anti-diabetic medication, %						<0.0001
Yes	8.38	0	0	57.88	68.2	
No	91.62	100	100	42.12	31.8	
Lipid-lowering agents, %						<0.0001
Yes	17.86	10.74	27.55	49.05	55.99	
No	82.14	89.26	72.45	50.95	44.01	
Anti-hypertensive medication, %						<0.0001
Yes	26.63	17.02	47.97	60.82	76.52	
No	73.37	82.98	52.03	39.18	23.48	
Aspirin, %						<0.0001
Yes	0.76	0.34	1.25	2.38	3.56	
No	99.24	99.66	98.75	97.62	96.44	
ACEI or ARB, %						<0.0001
Yes	17.45	10.05	29.03	47.21	59.06	
No	82.55	89.95	70.97	52.79	40.94	

Data are mean with SEMs; GFR is calculated using the CKD-EPI Creatinine Equation (2009). NLR: neutrophil to lymphocyte ratio; SII: systemic immune-inflammation index; SII = (Platelets × Neutrophil)/Lymphocytes; BMI: Body mass index; WBC: white blood cell; Hb, hemoglobin; PLT: platelet; UACR: urine albumin-creatinine ratio; ACEI: angiotensin-converting enzyme inhibitors; ARB: angiotensin II receptor blockers. CKD was classified according to the KDIGO guideline. *p* values are for comparisons using the ANOVA for continuous variables with a normal distribution and the Kruskal–Wallis test for continuous variables with a skewed distribution.

**Table 2:** Baseline raw correlation between neutrophils, neutrophil to lymphocyte ratio, systemic immune-inflammation index, and renal parameters.

	eGFR		UACR	
Variable	r (95% CI)	*p*	r (95% CI)	*p*
Neutrophils (×10^9^/L)	0.09(−0.69, 0.88)	0.81	33.93(9.05, 58.81)	0.01
Neutrophils percentage (%)	−0.36(−0.51, −0.21)	<0.0001	5.23(1.09, 9.36)	0.01
NLR	−3.16(−4.05, −2.26)	<0.0001	25.70(0.43, 50.98)	0.05
SII	−0.01(−0.01, 0.00)	<0.001	0.16(0.03, 0.29)	0.02

Data are mean with SEMs; GFR is calculated using the CKD-EPI Creatinine Equation (2009); NLR: neutrophil to lymphocyte ratio; SII: systemic immune-inflammation index; SII = (Platelets × Neutrophil)/Lymphocytes; CKD was classified according to the KDIGO guideline. *p* values are for comparisons using the ANOVA for continuous variables with a normal distribution and the Kruskal–Wallis test for continuous variables with a skewed distribution.

**Table 3:** Association of neutrophil count level with kidney function in patients with diabetic kidney disease from the NHANES 2005–2020.

Kidney Function Measures	Neutrophils Count Level (×10^9^/L)							
	Quartile 1	Quartile 2		Quartile 3		Quartile 4		
UACR								
Character	β (95% CI)	β (95% CI)	*p*	β (95% CI)	*p*	β (95% CI)	*p*	*p* for trend
Crude model	reference	73.8(−7.35, 154.95)	0.07	100.62(8.91, 192.34)	0.03	189.47(79.97, 298.97)	<0.001	0.001
Model 1	reference	89.28(14.54, 164.02)	0.02	125.64(32.42, 218.85)	0.01	219.56(104.53, 334.59)	<0.001	<0.001
Model 2	reference	108.12(21.92, 194.32)	0.01	102.76(5.33, 200.20)	0.04	260.98(126.71, 395.24)	<0.001	<0.001
Model 3	reference	102.58(17.96, 187.20)	0.02	87.48(−6.82, 181.77)	0.07	251.33(118.70, 383.96)	<0.001	<0.001
eGFR								
Character	β (95% CI)	β (95% CI)	*p*	β (95% CI)	*p*	β (95% CI)	*p*	*p* for trend
Crude model	reference	2.26(−1.91, 6.42)	0.29	2.91(−0.71, 6.54)	0.11	0.96(−3.58, 5.50)	0.68	0.70
Model 1	reference	−0.44(−2.94, 2.07)	0.73	−1.08(−3.96, 1.80)	0.46	−3.77(−6.60, −0.93)	0.01	0.01
Model 2	reference	−1(−3.56, 1.57)	0.44	−1.96(−5.24, 1.33)	0.24	−4.78(−7.80, −1.75)	0.002	0.002
Model 3	reference	−0.87(−3.40, 1.66)	0.50	−1.62(−4.96, 1.72)	0.34	−4.56(−7.60, −1.52)	0.004	0.003

Data are *n* or weighted β (95% CI). GFR is calculated using the CKD-EPI Creatinine Equation (2009); *p* values are for comparisons using the ANOVA for continuous variables with a normal distribution and the Kruskal–Wallis test for continuous variables with a skewed distribution. Crude Model: not adjusted; Model 1: adjusted age, sex, and race; Model 2: adjusted Model 1 + smoking + alcohol use; Model 3: adjusted Model 2 + hypertension + hyperlipidemia. Neutrophil count (×10^9^) was quartered: Q1 (∞, 3.5]; Q2 (3.5,4.5]; Q3 (4.5,5.6]; Q4 (5.6, ∞).

**Table 4:** Associations of neutrophil count level with all-cause and CVD mortality in patients with diabetic kidney disease from the NHANES 2005–2018 cohort.

		Crude Model		Model 1		Model 2		Model 3	
	No. Death/No at Risk	HR (95% CI)	*p*	HR (95% CI)	*p*	HR (95% CI)	*p*	HR (95% CI)	*p*
All-cause mortality									
Quartile 1	194/398	1(reference)		1(reference)		1(reference)		1(reference)	
Quartile 2	175/392	0.91(0.71,1.14)	0.40	0.92(0.73,1.15)	0.46	0.88(0.70,1.12)	0.31	0.88(0.70,1.11)	0.29
Quartile 3	178/333	1.13(0.87,1.47)	0.35	1.24(0.97,1.59)	0.08	1.21(0.94,1.57)	0.13	1.20(0.93,1.54)	0.15
Quartile 4	228/322	1.62(1.28,2.04)	<0.0001	1.98(1.55,2.53)	<0.0001	1.73(1.33,2.26)	<0.0001	1.73(1.34,2.25)	<0.0001
*p* for trend			<0.0001		<0.0001		<0.0001		<0.0001
Cardiovascular mortality									
Quartile 1	61/398	1(reference)		1(reference)		1(reference)		1(reference)	
Quartile 2	52/392	0.86(0.55,1.33)	0.49	0.88(0.55,1.38)	0.57	0.86(0.54,1.37)	0.52	0.83(0.52,1.32)	0.47
Quartile 3	58/333	1.16(0.76,1.79)	0.49	1.29(0.83,2.03)	0.26	1.27(0.81,2.00)	0.30	1.23(0.78,1.94)	0.35
Quartile 4	67/322	1.71(1.09,2.67)	0.02	2.25(1.42,3.57)	<0.001	1.82(1.13,2.94)	0.01	1.81(1.14,2.89)	0.01
*p* for trend			0.01		<0.001		0.01		0.004

Data are *n* or weighted HR (95% CI). GFR is calculated using the CKD-EPI Creatinine Equation (2009); *p* values are for comparisons using the ANOVA for continuous variables with a normal distribution and the Kruskal–Wallis test for continuous variables with a skewed distribution. Crude Model: no adjusted; Model 1: adjusted age, sex, and race; Model 2: adjusted Model 1 + smoking + alcohol use + hypertension + hyperlipidemia + eGFR + UACR; Model 3: adjusted Model 2 + aspirin. Neutrophil count (×10^9^) was quartered: Q1 (∞, 3.5]; Q2 (3.5,4.5]; Q3 (4.5,5.6]; Q4 (5.6, ∞).

## Data Availability

The data were all acquired from the NHANES database.

## Supplementary Materials

The following supporting information can be downloaded at: https://www.mdpi.com/article/10.3390/biomedicines12081907/s1, Table S1: Clinical characteristics of patients with different quartiles of circulating neutrophils count; Table S2: Clinical characteristics of patients in the development and validation cohorts used for the CVD risk prediction model; Table S3: CVD mortality-free survival among patients diagnosed with DKD in the development cohort between 2005 and 2018; Figure S4: The calibration plots for predicting the 5- and 10-year CVD mortality-free survival among DKD patients in the (A&B) development cohort and (C&D) validation cohorts; Table S5: Clinical characteristics of patients in the development and validation cohorts used for the risk prediction model for all-cause mortality; Table S6: Predictors for all-cause mortality in patients with diabetic kidney disease; Figure S7: Clinical predictive models for long-term survivals among individuals with DKD. (A) Nomogram for predicting 5- and 10-year overall survival of DKD patients in the development cohort. ROC curves of the predictive nomogram in (B) development and (C) validation cohorts; Figure S8: Overall survival (OS) in DKD patients with high-/low-risk scores and baseline neutrophil counts in the (A,B) development and (C,D) validation cohort; Figure S9: Clinical predictive models for long-term CVD mortality-free survival among individuals with DKD. (A) Nomogram for predicting 5- and 10-year CVD-mortality free survival between DKD patients in the development cohort. ROC curves of the predictive nomogram in (B) development and (C) validation cohorts. Q1, Quartile 1; Q2, Quartile 2; Q3, Quartile 3; Q4, Quartile 4.; Figure S10: CVD-free mortality in patients with diabetic kidney disease according to risk scores and baseline neutrophils counts. (A&B) Development cohort and (C&D) Validation cohort.
