# Supplementary material for: Higher Circulating Neutrophil Counts Is Associated with Increased Risk of All-Cause Mortality and Cardiovascular Disease in Patients with Diabetic Kidney Disease
Source: Biomedicines. 2024 Aug 20;12(8):1907. doi: 10.3390/biomedicines12081907 (PMC11352130; doi:10.3390/biomedicines12081907)
Supplement: Supplementary file 1 [file biomedicines-12-01907-s001.zip › biomedicines-3157472-supplementary.pdf]

Supplementary Material

S1. Clinical characteristics of patients with different quartiles of circulating neutrophils count

Variable	Total	Neutrophil Counts (×10⁹/L)				*P* value
		Q1	Q2	Q3	Q4	
		(∞,3.5]	(3.5,4.5]	(4.5,5.6]	(5.6, ∞)	
**Age**	64.50(63.70,65.29)	66.21(64.82,67.59)	65.13(63.47,66.78)	64.18(62.81,65.55)	62.95(61.32,64.57)	0.02
**Sex, %**						0.04
**Female**	49.53	46.15	46.69	47.6	56.21	
**Male**	50.47	53.85	53.31	52.4	43.79	
**Race, %**						< 0.0001
**White**	63.58	50.02	62.69	67.4	71.14	
**Black**	14.36	27.89	13.96	10.11	8.32	
**Mexican America**	9.82	9.70	10.1	10.68	8.94	
**Other**	12.24	12.4	13.25	11.8	11.61	
**Smoking, %**						< 0.0001
**Yes**	15	11.06	11.12	14.01	22.2	
**No**	85	88.94	88.88	85.99	77.8	
**Alcohol use, %**						0.35
**Yes**	55.13	51.2	57.13	57.76	54.03	
**No**	44.87	48.8	42.87	42.24	45.97	
**Hypertension, %**						0.90
**Yes**	81.86	80.98	82.81	82.51	81.12	
**No**	18.14	19.02	17.19	17.49	18.88	
**Hyperlipidemia, %**						0.17

**Yes**	89.97	88.91	87.63	92.78	90.39	
**No**	10.03	11.09	12.37	7.22	9.61	
**WBC (×10⁹/L)**	8.03(7.87,8.18)	5.57(5.39, 5.74)	6.94(6.83, 7.04)	8.17(8.03, 8.30)	10.69(10.40,10.98)	< 0.0001
**Neutrophils percentage (%)**	61.38(60.82,61.93)	52.65(51.74,53.56)	59.15(58.42,59.89)	63.04(62.13,63.95)	68.39(67.34,69.44)	< 0.0001
**Lymphocytes (×10⁹/L)**	2.14(2.05,2.23)	1.94(1.79,2.09)	2.04(1.94,2.13)	2.14(2.03,2.25)	2.38(2.15,2.62)	0.01
**Albumin, urine (mg/L)**	296.88(255.33,338.43)	213.34(173.49,253.20)	282.70(192.26,373.15)	253.51(179.29,327.72)	408.87(290.61,527.13)	0.01
**Creatinine, urine (mg/dL)**	111.93(108.16,115.69)	118.74(110.56,126.93)	110.89(104.19,117.59)	113.11(105.09,121.14)	106.75(100.10,113.40)	0.16
**UACR (mg/g)**	312.67(267.33,358.00)	209.64(161.57,257.71)	285.05(199.68,370.42)	280.13(185.62,374.65)	441.58(315.15,568.01)	0.01
**eGFR (ml/min/1.73m²)**	69.42(67.77,71.08)	69.05(66.08,72.01)	69.42(66.17,72.66)	69.94(66.53,73.34)	69.27(65.61,72.93)	0.98
**Anti-diabetic medication, %**						0.35
**Yes**	66.44	64.67	63.56	67.31	69.54	
**No**	33.56	35.33	36.44	32.69	30.46	
**Lipid-lowering agents, %**						0.88
**Yes**	55.21	54.4	53.88	55.39	56.82	
**No**	44.79	45.6	46.12	44.61	43.18	
**Anti-hypertensive medication, %**						0.32
**Yes**	76.69	75.11	75.64	80.97	75.09	
**No**	23.31	24.89	24.36	19.03	24.91	
**Aspirin, %**						0.08
**Yes**	3.87	5.7	4.47	2.58	3.11	
**No**	96.13	94.3	95.53	97.42	96.89	
**ACEI or ARB, %**						0.2
**Yes**	59.28	58.26	61.59	62.16	55.52	
**No**	40.72	41.74	38.41	37.84	44.48	

Data are mean with SEMs; GFR is calculated using the CKD-EPI Creatinine Equation (2009).

WBC: white blood cell; UACR: urine albumin-creatinine ratio; ACEI: angiotensin-converting enzyme inhibitors; ARB: angiotensin II receptor blockers.

*P values are for comparisons using the ANOVA for continuous variables with a normal distribution, the Kruskal-Wallis test for continuous variables with a skewed distribution.

S2. Clinical characteristics of patients in the development and validation cohorts used for the CVD risk prediction model.

Variable	Total (n=1683)	Development cohort (n=1179)	Validation cohort (n=504)	*P* value*
**Age**	62.76 (61.81,63.70)	63.08 (62.00,64.15)	62.06 (60.64,63.49)	0.21
**Sex, %**				1
**Female**	49.26	49.26	49.26	
**Male**	50.74	50.74	50.74	
**Race, %**				0.88
**White**	60.87	60.85	60.9	
**Black**	14.76	15.07	14.07	
**Mexican America**	11.11	10.82	11.73	
**Other**	13.27	13.26	13.3	
**Smoking, %**				0.35
**Yes**	14.48	15.28	12.72	
**No**	85.52	84.72	87.28	
**Alcohol use, %**				0.33
**Yes**	58.57	59.73	56.01	
**No**	41.43	40.27	43.99	
**Hypertension, %**				0.90
**Yes**	79.94	80.07	79.63	
**No**	20.06	19.93	20.37	
**Hyperlipidemia, %**				0.83
**Yes**	90.39	90.24	90.71	
**No**	9.61	9.76	9.29	
**Neutrophils count ($\times 10^9$/L)**	4.89 (4.78,5.00)	4.88 (4.73,5.02)	4.92 (4.71,5.14)	0.75
**UACR (mg/g)**	281.30 (231.47,331.13)	271.43 (213.27,329.59)	303.01 (199.94,406.07)	0.61
**eGFR (ml/min/1.73m^2)**	72.67 (70.79,74.56)	71.98 (69.77,74.18)	74.20 (70.67,77.73)	0.29
**Aspirin, %**				0.62
**Yes**	3.16	2.96	3.6	
**No**	96.84	97.04	96.4	
**Follow-up time (months)**	78.40 (75.19,81.61)	79.70 (76.79,82.62)	75.53 (69.34,81.72)	0.18

Data are n or weighted HR (95% CI). GFR is calculated using the CKD-EPI Creatinine Equation (2009); P values are for comparisons using the ANOVA for continuous variables with a normal distribution, the Kruskal-Wallis test for continuous variables with a skewed distribution.

S3. CVD mortality-free survival among patients diagnosed with DKD in the development cohort between 2005 and 2018.

	Univariate Analysis		Multivariate Analysis	
**Covariate**	**HR (95% CI)**	**P value**	**HR (95% CI)**	**P value**
**Age**				
**< 50**				
**50-59**	1.94 (0.58, 6.42)	0.28	1.91 (0.57, 6.37)	0.29
**60-69**	3.32 (1.16, 9.47)	0.025	3.34 (1.16, 9.66)	0.026
**70-79**	9.04 (3.28, 24.92)	2.09E-05	9.61 (3.36, 27.49)	2.45E-05
**≥ 80**	25.05 (9.11, 68.90)	4.43E-10	27.58 (9.38, 81.06)	1.64E-09
**Sex (male *v* female)**	1.08 (0.79, 1.47)	0.63	1.32 (0.95, 1.83)	0.10
**Race**				
**White**				
**Black**	0.57 (0.39, 0.82)	0.0029	0.88 (0.58, 1.31)	0.52
**Mexican America**	0.34 (0.20, 0.56)	2.65E-05	0.60 (0.35, 1.01)	0.055
**Other**	0.38 (0.22, 0.64)	3.43E-04	0.52 (0.30, 0.91)	0.021
**Smoking (yes *v* no)**	0.90 (0.59, 1.37)	0.63	2.07 (1.29, 3.31)	0.0027
**Alcohol use (yes *v* no)**	0.57 (0.41, 0.78)	0.00054	0.70 (0.50, 0.99)	0.041
**Neutrophils count ($\times 10^9$/L)**				
**Q1**				
**Q2**	0.95 (0.61, 1.48)	0.82	1.05 (0.67, 1.65)	0.82
**Q3**	1.10 (0.726, 1.70)	0.65	1.29 (0.83, 2.00)	0.27
**Q4**	1.50 (0.99, 2.28)	0.057	1.80 (1.15, 2.84)	0.011
**Hypertension (yes *vs* no)**	1.44 (0.93, 2.22)	0.10	1.13 (0.72, 1.77)	0.60
**Hyperlipidemia (yes *vs* no)**	0.95 (0.57, 1.57)	0.83	0.97 (0.58, 1.62)	0.91
**UACR (mg/g)**				
**Normal**				
**Microalbumin**	0.74 (0.52, 1.07)	0.11	1.66 (1.09, 2.51)	0.017
**Macroalbumin**	1.36 (0.87, 2.11)	0.17	2.94 (1.77, 4.89)	3.10E-05
**eGFR (mL/min/1.73m^2)**				
**≥ 60**				
**30-59**	2.83 (2.02, 3.97)	1.42E-09	1.66 (1.06, 2.49)	0.026
**< 30**	4.18 (2.27, 7.69)	4.42E-06	2.94 (0.94, 3.59)	0.075

Data are n or weighted HR (95% CI). GFR is calculated using the CKD-EPI Creatinine Equation (2009); P values are for comparisons using the ANOVA for continuous variables with a normal distribution, the Kruskal-Wallis test for continuous variables with a skewed distribution.

S4. The calibration plots for predicting the 5- and 10-year CVD mortality-free survival among DKD patients in the (A&B) development cohort and (C&D) validation cohorts.

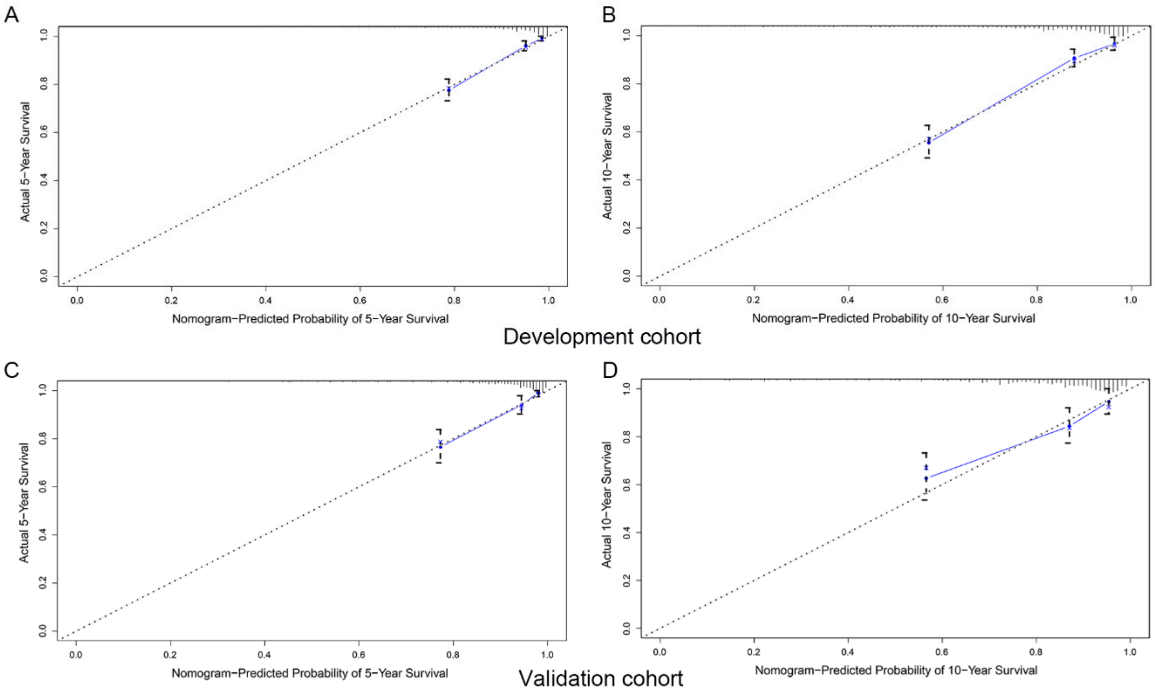

S5. Clinical characteristics of patients in the development and validation cohorts used for the risk prediction model for all-cause mortality.

Variable	Total (n=2220)	Development cohort (n=1556)	Validation cohort (n=664)	*P* value*
**Age**	64.50 (63.70,65.29)	64.49 (63.67,65.32)	64.50 (63.13,65.88)	0.99
**Sex, %**				0.22
**Female**	49.53	48.45	52.17	
**Male**	50.47	51.55	47.83	
**Race, %**				0.84
**White**	63.58	63.18	64.54	
**Black**	14.36	14.25	14.64	
**Mexican America**	9.82	10.08	9.2	
**Other**	12.24	12.49	11.62	
**Smoking, %**				0.73
**Yes**	15	14.82	15.45	
**No**	85	85.18	84.55	
**Alcohol use, %**				0.63
**Yes**	55.13	54.71	56.15	
**No**	44.87	45.29	43.85	
**Hypertension, %**				0.7
**Yes**	81.86	82.15	81.15	
**No**	18.14	17.85	18.85	
**Hyperlipidemia, %**				0.38
**Yes**	89.97	90.46	88.79	
**No**	10.03	9.54	11.21	
**Neutrophils count ($\times 10^9$/L)**	4.97 (4.87,5.08)	4.95 (4.84,5.07)	5.03 (4.83,5.23)	0.48
**UACR (mg/g)**	312.67 (267.33,358.00)	319.33 (263.36,375.30)	296.44 (222.03,370.84)	0.63
**eGFR (ml/min/1.73m^2)**	69.42 (67.77,71.08)	70.27 (68.29,72.25)	67.37 (64.70,70.04)	0.08
**Aspirin, %**				0.88
**Yes**	3.87	3.83	3.98	
**No**	96.13	96.17	96.02	
**Follow-up time (months)**	74.17 (71.29,77.04)	73.94 (70.76,77.11)	74.72 (70.06,79.39)	0.76

Data are n or weighted HR (95% CI). GFR is calculated using the CKD-EPI Creatinine Equation (2009); P values are for comparisons using the ANOVA for continuous variables with a normal distribution, the Kruskal-Wallis test for continuous variables with a skewed distribution.

S6. Predictors for all-cause mortality in patients with diabetic kidney disease

	Univariate Analysis		Multivariate Analysis	
**Covariate**	**HR (95% CI)**	***P* value**	**HR (95% CI)**	***P* value**
**Age**				
**< 50**				
**50-59**	1.55 (0.93, 2.59)	0.094	1.55 (0.92, 2.60)	0.010
**60-69**	2.22 (1.43, 3.44)	3.75E-06	2.18 (1.39, 3.41)	6.50E-04
**70-79**	4.23 (2.77, 6.48)	2.99E-11	4.47 (2.85, 7.01)	7.19E-11
**≥ 80**	9.65 (6.29, 14.81)	< 2E-16	10.13 (6.34, 16.17)	< 2E-16
**Sex (male *v* female)**	1.23 (1.04, 1.46)	0.014	1.44 (1.12, 1.60)	0.0013
**Race**				
**White**				
**Black**	0.61 (0.50, 0.76)	5.55E-06	0.92 (0.73, 1.15)	0.45
**Mexican America**	0.45 (0.35, 0.58)	1.82E-09	0.70 (0.53, 0.92)	0.011
**Other**	0.53 (0.40, 0.70)	3.82E-06	0.77 (0.59, 1.02)	0.071
**Smoking (yes *v* no)**	1.12 (0.90, 1.40)	0.29	1.90 (1.49, 2.42)	2.34E-07
**Alcohol use (yes *v* no)**	0.64 (0.54, 0.77)	6.33E-07	0.74 (0.61, 0.88)	0.0010
**Neutrophils count ($\times 10^9$/L)**				
**Q1**				
**Q2**	1.09 (0.86, 1.40)	0.47	1.14 (0.90, 1.46)	0.28
**Q3**	1.14 (0.89, 1.45)	0.29	1.19 (0.93, 1.52)	0.18
**Q4**	1.65 (1.31, 1.97)	2.04E-05	1.67 (1.31, 2.13)	3.49E-05
**Hypertension (yes *v* no)**	1.47 (1.15, 1.87)	0.0021	1.14 (0.87, 1.47)	0.31
**Hyperlipidemia (yes *v* no)**	0.80 (0.62, 1.03)	0.087	0.88 (0.68, 1.14)	0.33
**UACR (mg/g)**				
**Normal**				
**Microalbuminuria**	0.90 (0.73, 1.10)	0.29	1.61 (1.27, 2.05)	0.00010
**Macroalbuminuria**	1.42 (1.12, 1.81)	0.0044	2.51 (1.89, 3.31)	1.19E-10
**eGFR (mL/min/1.73m^2)**				
**≥ 60**				
**30-59**	1.88 (1.57, 2.25)	3.5E-12	1.46 (1.17, 1.83)	0.0061
**< 30**	3.06 (2.25, 4.15)	9.5E-13	1.72 (1.24, 2.39)	0.00049

Data are n or weighted HR (95% CI). GFR is calculated using the CKD-EPI Creatinine Equation (2009); P values are for comparisons using the ANOVA for continuous variables with a normal distribution, the Kruskal-Wallis test for continuous variables with a skewed distribution.

S7. Clinical predictive models for long-term survivals among individuals with DKD. (A) Nomogram for predicting 5- and 10-year overall survival of DKD patients in the development cohort. ROC curves of the predictive nomogram in (B) development and (C) validation cohorts.

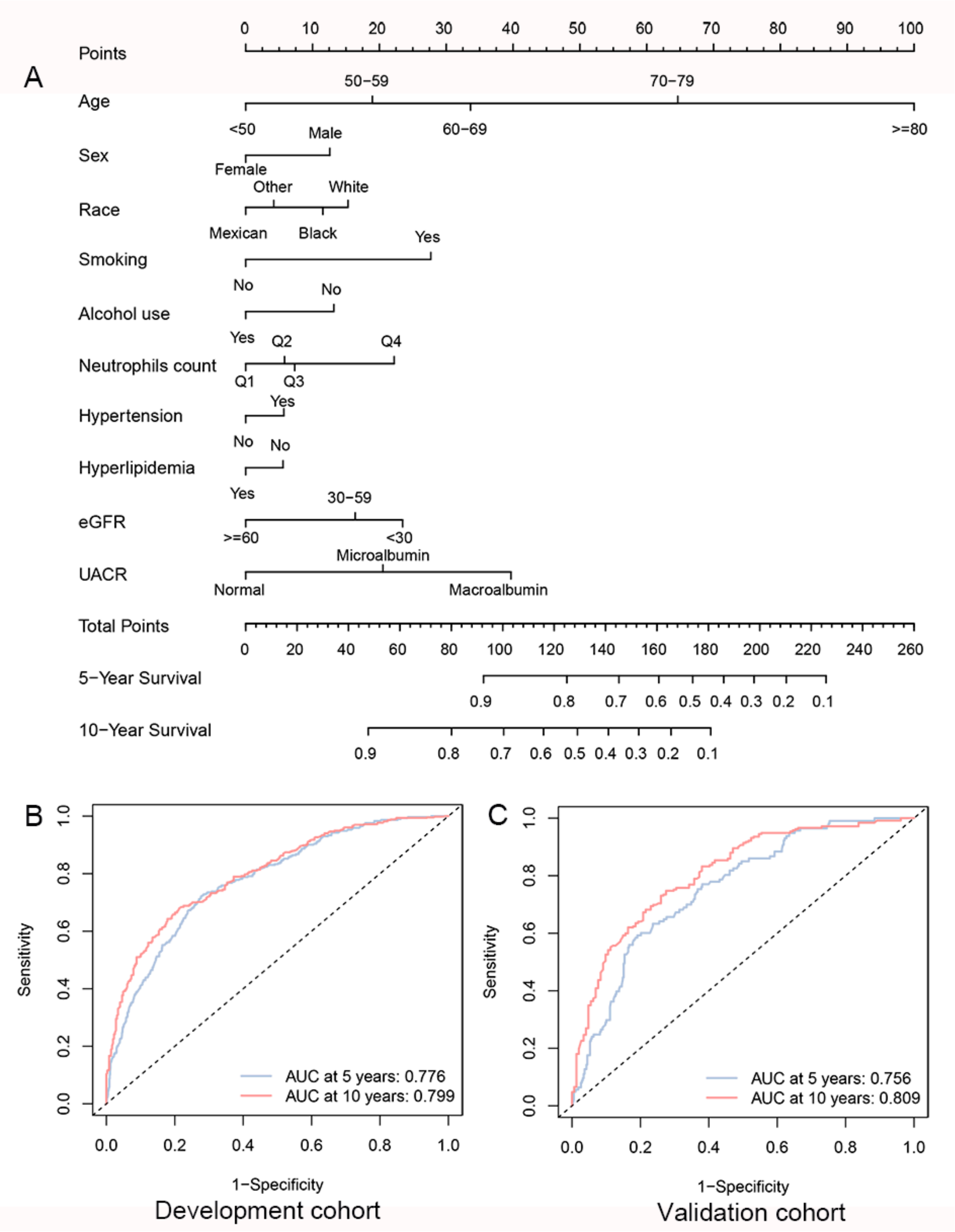

S8. Overall survival (OS) in DKD patients with high-/low-risk scores and baseline neutrophil counts in the (A-B) development and (C-D) validation cohort.

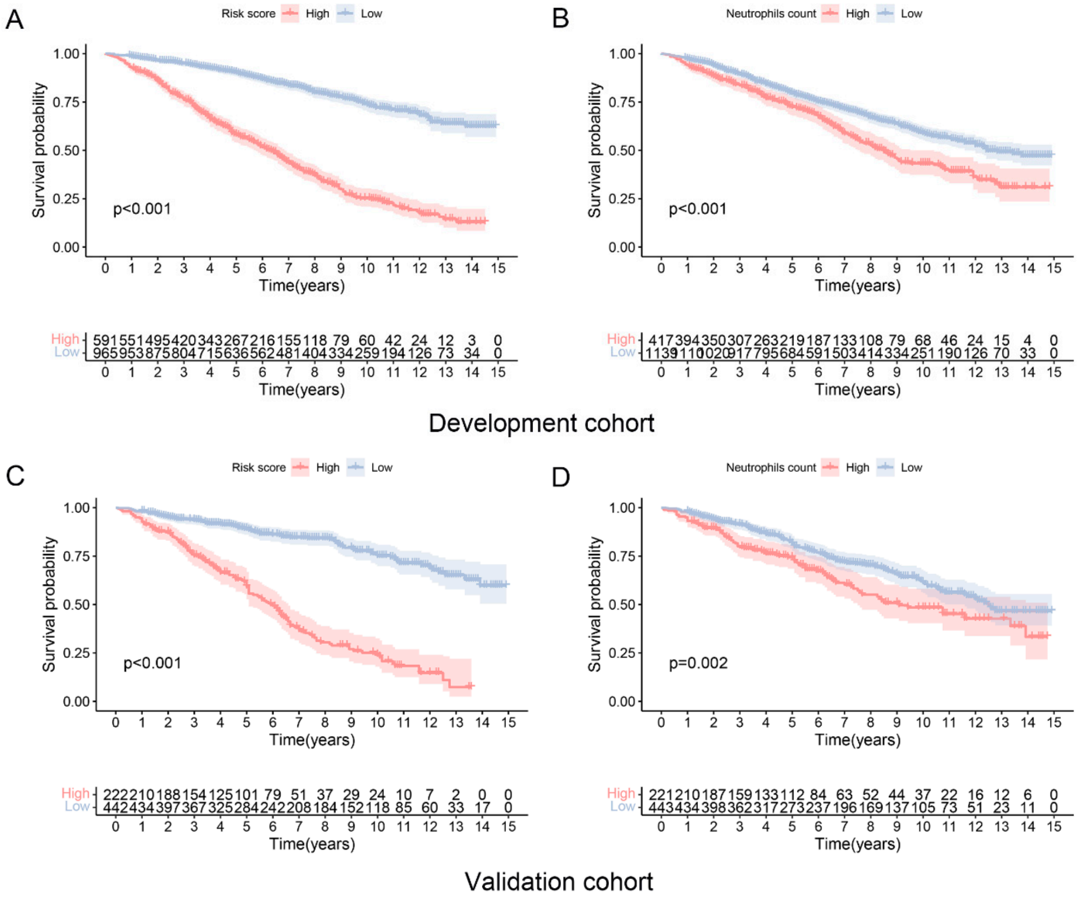

S9. Clinical predictive models for long-term CVD mortality-free survival among individuals with DKD. (A) Nomogram for predicting 5- and 10-year CVD-mortality free survival between DKD patients in the development cohort. ROC curves of the predictive nomogram in (B) development and (C) validation cohorts. Q1, Quartile 1; Q2, Quartile 2; Q3, Quartile 3; Q4, Quartile 4.

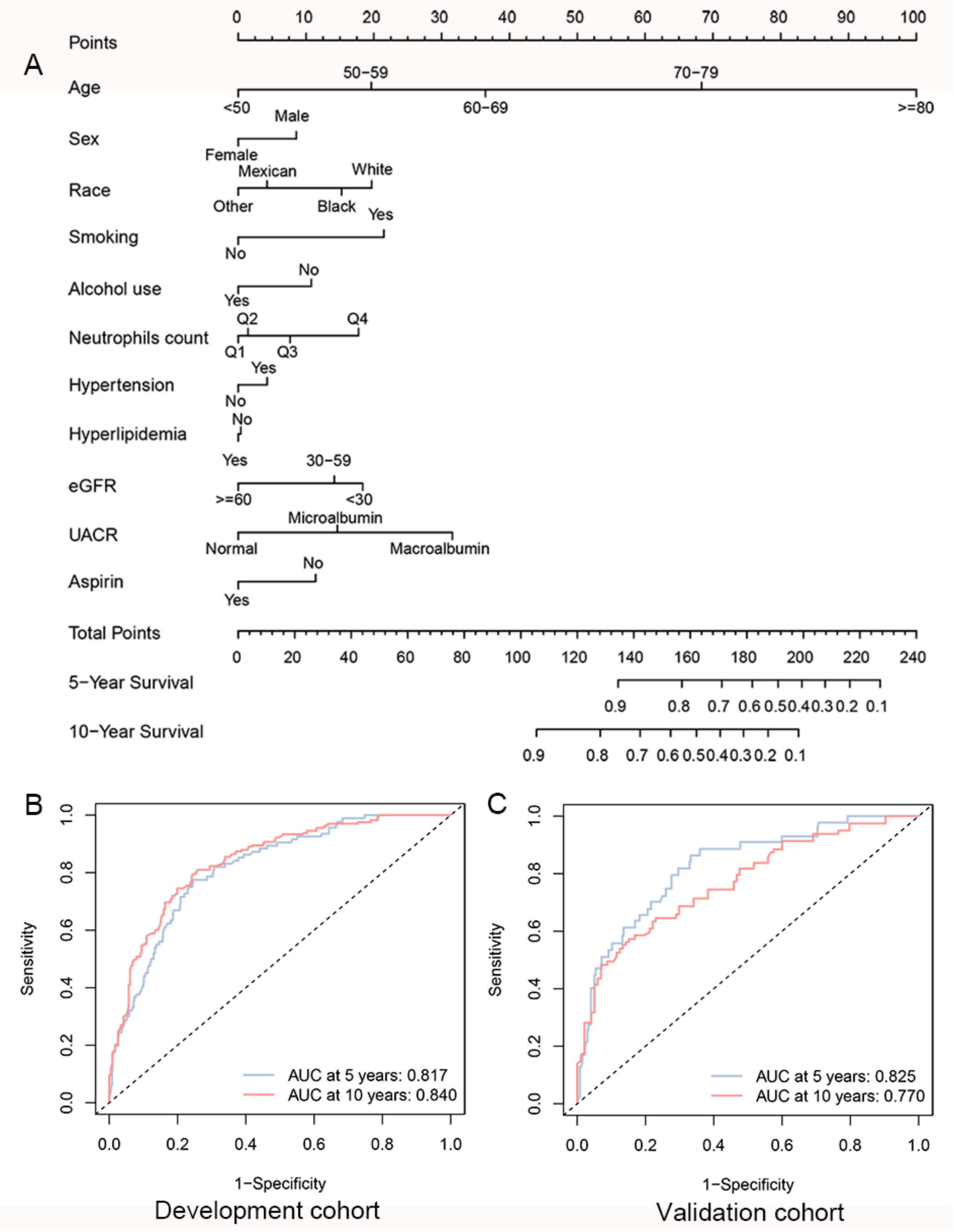

* Model was also adjusted for the use of aspirin

S10. CVD-free mortality in patients with diabetic kidney disease according to risk scores and baseline neutrophils counts. (A&B) Development cohort and (C&D) Validation cohort.

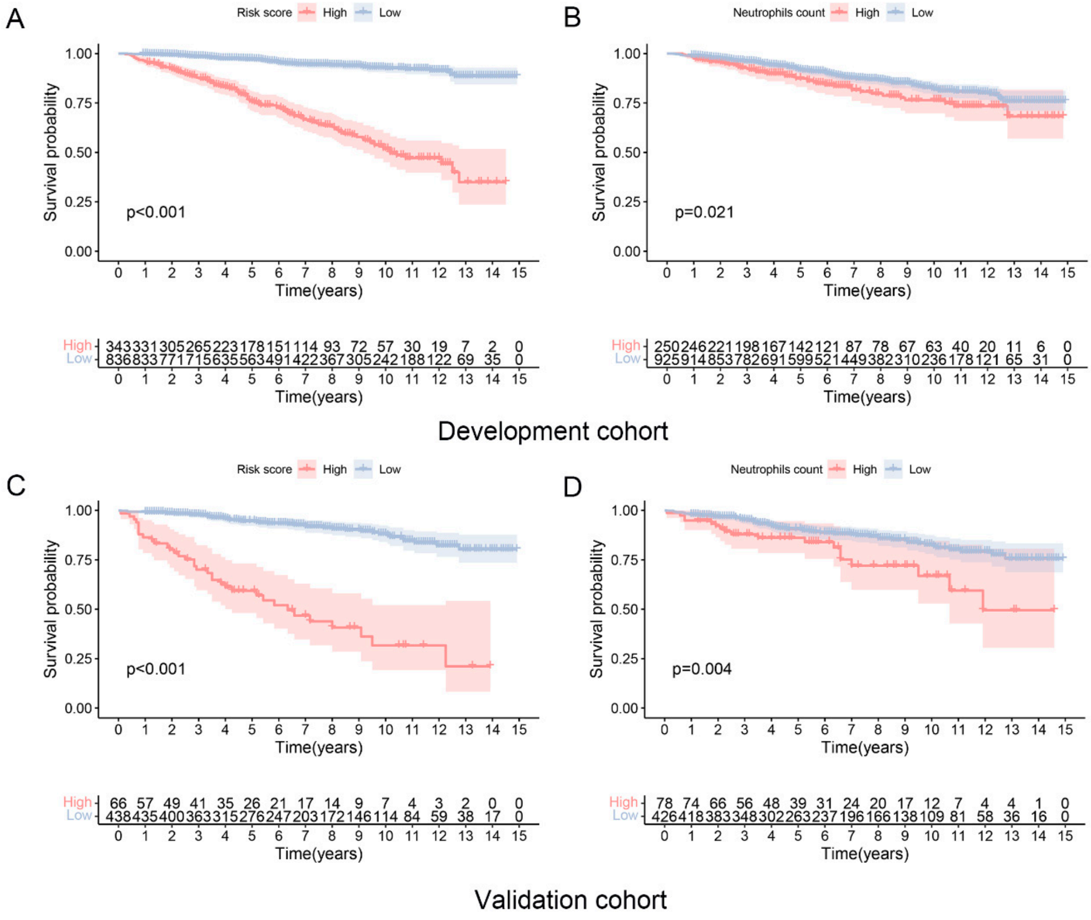

*Model was also adjusted for the use of aspirin
